# The Role of NRF2 in Trinucleotide Repeat Expansion Disorders

**DOI:** 10.3390/antiox13060649

**Published:** 2024-05-26

**Authors:** Kuo-Hsuan Chang, Chiung-Mei Chen

**Affiliations:** 1 Department of Neurology, Chang Gung Memorial Hospital, Linkou Medical Center, Kueishan, Taoyuan 333, Taiwan; gophy5128@cgmh.org.tw; 2 College of Medicine, Chang Gung University, Taoyuan 333, Taiwan

**Keywords:** trinucleotide repeat expansion disorders, neurodegeneration, oxidative stress, NRF2, anti-oxidative therapy

## Abstract

Trinucleotide repeat expansion disorders, a diverse group of neurodegenerative diseases, are caused by abnormal expansions within specific genes. These expansions trigger a cascade of cellular damage, including protein aggregation and abnormal RNA binding. A key contributor to this damage is oxidative stress, an imbalance of reactive oxygen species that harms cellular components. This review explores the interplay between oxidative stress and the NRF2 pathway in these disorders. NRF2 acts as the master regulator of the cellular antioxidant response, orchestrating the expression of enzymes that combat oxidative stress. Trinucleotide repeat expansion disorders often exhibit impaired NRF2 signaling, resulting in inadequate responses to excessive ROS production. NRF2 activation has been shown to upregulate antioxidative gene expression, effectively alleviating oxidative stress damage. NRF2 activators, such as omaveloxolone, vatiquinone, curcumin, sulforaphane, dimethyl fumarate, and resveratrol, demonstrate neuroprotective effects by reducing oxidative stress in experimental cell and animal models of these diseases. However, translating these findings into successful clinical applications requires further research. In this article, we review the literature supporting the role of NRF2 in the pathogenesis of these diseases and the potential therapeutics of NRF2 activators.

## 1. Introduction

Microsatellites are short, repetitive sequences of DNA found throughout the genomes of both prokaryotes and eukaryotes [1,2]. They consist of 1–6 base pairs, mostly repeated 20–100 times, and can be found in both protein-coding and non-coding regions [3]. These microsatellites play various roles in biological regulation, influencing processes like alternative splicing, transcription initiation and termination, and DNA packaging [4,5,6,7,8,9]. One of the most striking features of microsatellites is their high mutation rate, which occurs 100 to a million times more frequently than that in non-repetitive regions [10,11]. This instability causes high variability in the number of repeats across individuals. This characteristic, however, also contributes to the development of various neurological diseases, including trinucleotide repeat expansion disorders.

Trinucleotide repeat disorders are a group of neuropsychiatric diseases caused by abnormal expansions of specific three-nucleotide microsatellites (Table 1). An understanding of the disorders began with the identification of (CGG)n trinucleotide repeats within the 5′ untranslated region (UTR) of FMR1 gene in Fragile X syndrome [12,13,14,15] and (CAG)n trinucleotide repeats within the coding region of the androgen receptor (AR) gene in spinobulbar muscular atrophy [16]. Further research revealed that similar expansions in other genes, particularly those encoding proteins with polyglutamine tracts, contribute to various neurodegenerative diseases such as Huntington disease (HD) [17], dentatorubropallidoluysian atrophy (DRPLA), and spinocerebellar atrophy (SCA) 1, 2, 3, 6, 7 and 17 [18]. Furthermore, more neurological disorders were identified as being caused by untranslated trinucleotide repeats located at 5′UTR for SCA12 [19], in the intronic regions for Friedreich ataxia [20], and in potential antisense sequences for SCA8 [21]. These diseases exhibit “intergenerational anticipation”, where symptoms appear earlier in offspring due to the expansions growing larger with each generation [22,23]. The instability of trinucleotide repeats can also be increased by various environmental stressors, such as cold, heat, hypoxic, and oxidative stresses, and also because specific repair pathways are involved in their repair [24,25]. The pathogenic expansions can range from a few repeats to thousands, while the severity of the disease phenotype often correlates directly with the length of the repeat expansions, with longer expansions leading to more severe symptoms [22,23]. Patients can exhibit varying symptoms and co-morbidities, depending on the specific length of their expansions within the same disease-causing gene [26,27,28,29,30,31,32].

The pathogenesis of trinucleotide repeat expansion leads to structural alterations in DNA, triggering a cascade of molecular processes affecting DNA, RNA, and protein levels [33,34,35]. The expanded polyglutamine tracts in HD, SCA and DRPLA exert a toxic effect by causing aberrant nuclear and cytoplasmic protein aggregation and by trapping transcription factors, chaperons, and proteins belonging to the ubiquitin–proteasome system (UPS) [36,37,38]. RNA transcripts with expanded repeats can bind RNA-binding proteins, altering their activity and location [39,40,41,42]. Expanded repeats can be translated, either through traditional AUG initiation, near-start codons, or a unique process called repeat-associated non-AUG (RAN) translation to generate proteins containing a pathogenic stretch of repeated amino acids [43].

Oxidative stress, an imbalance between the production and elimination of reactive oxygen species (ROS) in cells, can damage lipids, proteins, and nucleic acids, leading to cell death [44,45]. Both external factors, like UV light, and internal factors, like mitochondrial activity, contribute to ROS production [46,47,48]. While these oxygen byproducts play a role in cellular signaling, their excessive accumulation requires an efficient antioxidant defense system [49,50,51,52]. This system includes enzymes like superoxide dismutases (SODs) and catalase (CAT), as well as scavenger molecules like glutathione (GSH) [53,54,55]. Notably, GSH is a crucial antioxidant, directly scavenging ROS and acting as a cofactor for other antioxidant enzymes and detoxification processes [56,57,58,59]. Its synthesis is a two-step process involving glutamate cysteine ligase (GCL) and glutathione synthetase (GSS) [60]. Under normal conditions, cells maintain a basal level of antioxidant machinery. However, upon encountering oxidative stress, cells activate the nuclear factor–erythroid factor 2 (NRF2) pathway to further boost the expression of antioxidant enzymes, GSH synthesis, and detoxification enzymes, ensuring a robust protective response [61,62,63,64]. Interestingly, oxidative stress has been linked to various neurodegenerative diseases, including those caused by trinucleotide repeat expansions [65,66]. This review summarizes the latest evidence connecting oxidative stress to these disorders, with a specific focus on the NRF2 signaling pathway, which plays a critical role in regulating the cellular antioxidant response and may offer insights into potential therapeutic strategies.

## 2. Structure and Regulation of NRF2

**Figure 1:**
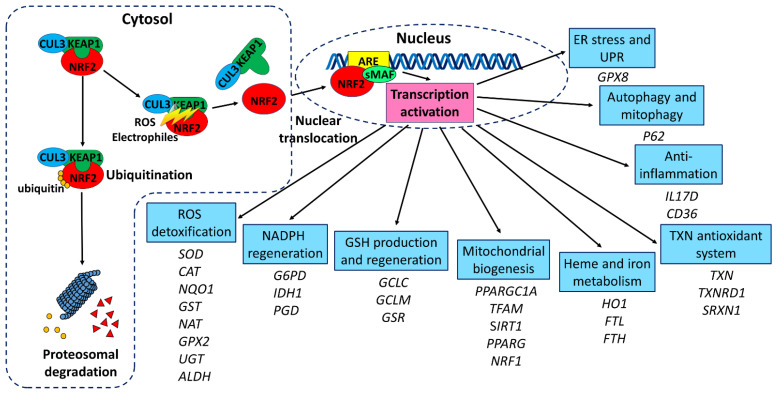
NRF2 activation and downstream cytoprotective responses. Under normal conditions, NRF2 binds to KEAP1, undergoing ubiquitination by the CUL3 (E3 ubiquitin ligase) and subsequent degradation by the proteasome in cytosol. When cells encounter ROS or electrophiles, NRF2 is dissociated from KEAP1 and translocates to the nucleus, where it forms heterodimers with sMAF. These complexes bind to the ARE, initiating the transcription of genes involved in ROS detoxification, NADPH regeneration, GSH production and regeneration, heme and iron metabolism, the TXN antioxidant system, and mitochondrial biogenesis. ALDH: aldehyde dehydrogenase; ARE: antioxidant response element; BCL2: B-cell lymphoma 2; CAT: catalase: CD36: cluster of differentiation 36; CUL3: cullin 3; FTH: ferritin heavy chain; ER: endoplasmic reticulum; FTL: ferritin light chain; G6PD: glucose-6-phosphate dehydrogenase; GCLC: glutamate-cysteine ligase catalytic subunit; GCLM: glutamate-cysteine ligase modifier subunit; GPX2: glutathione peroxidase 2; GPX8: glutathione peroxidase 8; GSR: glutathione reductase; GST: glutathione S-transferase; GSH: glutathione; GST: glutathione S-transferase; HO1: Heme oxygenase 1; IDH1: isocitrate dehydrogenase 1; KEAP1: Kelch-like ECH-associated protein 1; IL17D: interleukin-17D; sMAF: small musculoaponeurotic fibrosarcoma protein; NADPH: nicotinamide adenine dinucleotide phosphate; NAT: N-acetyltransferase; NQO1: NAD(P)H quinone dehydrogenase 1; NRF1: nuclear respiratory factor 1; NRF2: nuclear factor erythroid 2-related factor 2; PGD: 6-phosphogluconate dehydrogenase; PPARG: peroxisome proliferator-activated receptor γ; PPARGC1A: peroxisome proliferator-activated receptor γ coactivator 1-α; ROS: reactive oxygen species; SG Tang: SIRT1: sirtuin 1; SOD: superoxide dismutase; SRXN1: sulfiredoxin 1; TFAM: mitochondrial transcription factor A; TXN: thioredoxin; TXNRD1: thioredoxin reductase 1; UGT: UDP-glucuronosyltransferase. UPR: unfolded protein response.

NRF2 is a complex protein of 605 amino acids with 7 conserved NRF2-ECH homology (NEH) domains [67]. Under physiological conditions, NRF2 has a short half-life of only 15–40 min and is primarily located in the cytoplasm [68]. This tight regulation is achieved through the interaction between the NEH2 domain of NRF2 and the double-glycine repeat (DGR) region of Kelch-like ECH-associated protein 1 (KEAP1) [69]. The bric-a-brac, tramtrack, broad-complex (BTB) domain in KEAP1 mediates homodimerization and forms a complex with Cullin 3 (CUL3), RING-box adaptor 1 (RBX1), and an E2 ubiquitin ligase [70]. This KEAP1–CUL3–RBX1 complex acts as the primary regulator of NRF2 degradation by tagging it with ubiquitin for proteasomal degradation [70] (Figure 1).

**Figure 2:**
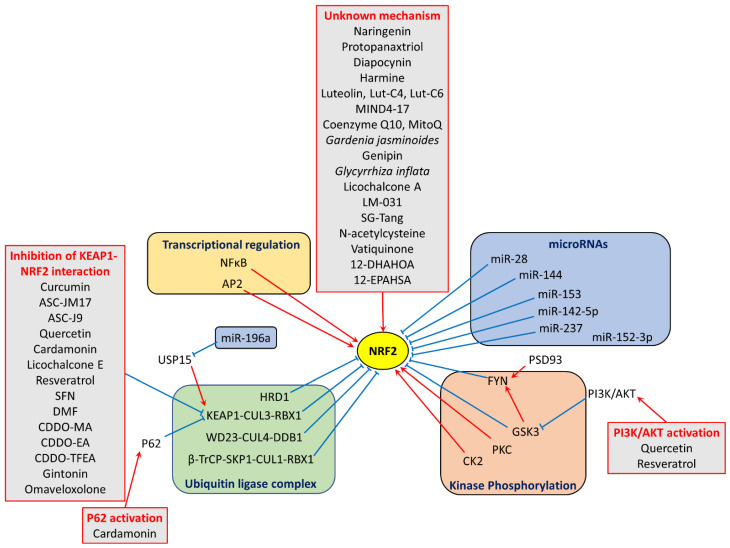
Regulation of NRF2 activity. Transcription factors like NF-κB and AP2 can activate NRF2 gene expression. MicroRNAs inhibit NRF2 protein production by binding to its messenger RNA. Post-translational regulation of NRF2 activity involves ubiquitin ligase complexes and kinase phosphorylation. Various compounds modulate NRF2 activity by inhibiting KEAP1-NRF2 interaction, activating P62, activating PI3K/AKT, and through mechanisms that remain unknown. Red arrows denote activation, while blue blunt ends represent inhibition. 12-DHAHOA: 12-docosahexaenoic acid hydroxy oleic acid; 12-EPAHSA: 12-eicosapentaenoic acid hydroxy stearic acid; AP2: activating protein 2; β-TrCP: β-transducin repeat-containing protein; CDDO-EA: 2-cyano-3,12-dioxooleana-1,9-dien-28-oic acid-ethyl amide; CDDO-MA: 2-cyano-3,12-dioxooleana-1,9-dien-28-oic acid-methyl amide; CDDP-TFMA: 2-cyano-3,12-dioxooleana-1,9-dien-28-oic acid-trifluoroethyl amide; CK2: casein kinase 2; CUL1: cullin 1; CUL3: cullin 3; CUL4: cullin 4; DDB1: damaged DNA binding protein 1; DMF: dimethyl fumarate; FYN: proto-oncogene tyrosine-protein kinase Fyn; GSK3: glycogen synthase kinase 3; HRD1: HMG-CoA reductase degradation protein 1; KEAP1: kelch-like ECH-associated protein 1; LM-031: 3-Benzoyl-5-Hydroxy-2H-Chromen-2-One; MIND4-17: 5-nitro-2-{[5-(phenoxymethyl)-4-phenyl-4H-1,2,4-triazol-3-yl]thio}pyridine; NFκB: nuclear factor kappa-light-chain-enhancer of activated B cells; NRF2: nuclear factor (erythroid-derived 2)-like 2; P62: sequestosome 1; PI3K/AKT: phosphatidylinositol 3-kinase/protein kinase B; PKC: protein kinase C; PSD93: postsynaptic density protein 93; RBX1: ring finger protein 1; SFN: sulforaphane; SG-Tang: Shaoyao Gancao Tang; SKP1: S-phase kinase-associated protein 1; USP15: ubiquitin-specific peptidase 15; WD23: WD-repeat protein 23.

Exposure to ROS, electrophiles, or heavy metals can covalently modify specific cysteine residues on KEAP1 and disrupt its attachment to NRF2 [71,72]. This allows NRF2 to avoid proteasomal degradation and translocate into the nucleus [73]. In the nucleus, the NEH1 domain of NRF2 binds to small, musculoaponeurotic fibrosarcoma proteins (sMAF) and antioxidant response element (ARE) on DNA [74,75,76], and initiates the transcription of genes that produce various protective molecules involving ROS detoxification, nicotinamide adenine dinucleotide phosphate (NADPH) regeneration, GSH production and regeneration, heme and iron metabolism, thioredoxin (TXN) antioxidant system, and mitochondrial biogenesis (Figure 1) [76]. The functional analysis of ARE identified a core sequence, 5′-TGACNNNGC-3′, which is essential for mediating basal and/or inducible activity [77]. The NEH3-5 acts as transactivation domains to further booster the transcription of several protective genes [78,79]. On the other hand, the NEH6 acts as a negative regulator, promoting the ubiquitination and subsequent degradation of NRF2 [80]. The NEH7 domain interacts with retinoic X receptor alpha (RXRa), a nuclear receptor that suppresses the NRF2/ARE pathway and reduces ARE gene expression [81]. In the cytoplasm, free NRF2 can be phosphorylated by GSK3β, leading to its increased degradation through the proteasomal pathway (Figure 2) [63,68].

NRF2 is encoded by the nuclear factor (erythroid-derived 2)-like 2 gene (*NFE2L2*) gene on chromosome 2q31.2 [82]. Two ARE sequences within the NFE2L2 promoter enable NRF2 to bind and enhance its own expression [83]. The presence of a nuclear factor kappa-light-chain-enhancer of the activated B cells (NFκB)-binding region in the *NFE2L2* promoter allows for the upregulation of NRF2 expression activated by NFκB during acute inflammation or tumorigenesis (Figure 2) [84]. The *NFE2L2* promoter also contains multiple binding sites for the activating enhancer-binding protein 2 (AP2) transcription factor, which interacts with a range of proteins as co-activators or suppressors of NRF2 transcription [85]. Interestingly, the *NFE2L2* promoter is rich in the CpG islands susceptible to methylation-induced silencing, highlighting the role of epigenetics in NFE2L2 regulation [86,87].

While the KEAP1–CUL3–RBX1 complex is a well-established regulator of NRF2 degradation, other E3 ubiquitin ligase complexes contribute to KEAP1-independent NRF2 degradation (Figure 2). The β-transducin repeat-containing protein (β-TrCP) binds to the NEH6 domain of NRF2, forming a ubiquitin ligase complex with S-phase kinase-associated protein-1 (SKP1), CUL1, and RBX1 for subsequent degradation of NRF2 [80]. WD-repeat protein 23 (WDR23) binds the NEH2 domain of NRF2 and interacts with the CUL4-damaged DNA-binding protein 1 (DDB1) ubiquitin ligase complex [80]. Another ubiquitin ligase, HMG-CoA reductase degradation 1 (HRD1), targets NRF2 for degradation by binding to its NEH4-5 domains, thereby attenuating NRF2 signaling [88]. NRF2 activity is also modulated by kinases that directly phosphorylate specific NRF2 domains. Protein kinase C (PKC) phosphorylates the NEH2 domain, preventing complex formation with KEAP1 and facilitating NRF2 translocation to the nucleus [89]. Casein kinase 2 (CK2) phosphorylates the NEH4-5 domains, leading to increased nuclear NRF2 levels [90]. The proto-oncogene tyrosine-protein kinase Fyn (FYN) phosphorylates the Y568 residue in the NEH1 domain, which triggers the export of NRF2 from the nucleus [91]. Glycogen synthase kinase 3 (GSK-3) phosphorylates the NEH6 domain to enhance the binding of β-TrCP to NRF2 to promote NRF2 degradation [92]. GSK-3also phosphorylates FYN, enabling its nuclear translocation, where it subsequently phosphorylates NRF2, leading to its nuclear export [91]. Interestingly, the phosphatidylinositol 3-kinase (PI3K)/protein kinase B (AKT) pathway promotes NRF2 activity by enhancing the inhibitory phosphorylation of GSK3 [92,93].

The expression of NRF2 is also controlled by microRNAs (Figure 2) [94,95,96,97]. For example, miR-144, miR-28, miR-142-5p, miR-153, and miR-237 directly target *NFE2L2* and downregulate NRF2 expression in various cell types [98,99,100]. In neurons, miR-152-3p protects against oxygen and glucose deprivation/reperfusion injury by enhancing NRF2/ARE signaling through the direct inhibition of postsynaptic density protein 93 (PSD93), an activator of FYN that promotes NRF2 nuclear export [101]. This complex system delicately regulates the response of NRF2 and ensures an appropriate level of protection against oxidative stress.

## 3. NRF2/ARE Pathway

Genome-wide studies have identified a vast network of genes regulated by ARE, providing insight into the diverse functions of NRF2 [102,103,104]. The core function among these target genes is the enhancement of resistance to oxidative stress. Moreover, NRF2 has been implicated in the regulation of inflammation, autophagy and mitophagy, as well as mitochondrial biogenesis.

### 3.1. Oxidative Stress

NRF2 plays a crucial role in orchestrating a comprehensive cellular defense against oxidative stress, initiating the transcription of numerous genes containing AREs. AREs were first discovered as cis-regulatory elements for NADPH quinone dehydrogenase 1 (*NQO1*) and glutathione S-transferase (*GST*) genes [105]. Subsequent investigations broadened the spectrum of proteins encoded by the ARE gene battery, encompassing the genes involved in drug detoxification, antioxidant responses, NADPH regeneration, and metabolic regulation [106]. In vivo studies using *NFE2L2* knockout mice demonstrate that NRF2 governs the expression of these antioxidant and cytoprotective genes [76,107]. NRF2 exerts control over key components of the GSH and TXN antioxidant systems, as well as enzymes implicated in NADPH regeneration, ROS detoxification, and heme metabolism, thereby playing a fundamental role in maintaining cellular redox homeostasis (Figure 1) [44]. Tight regulation of GSH levels by NRF2 involves direct control over the expression of glutathione reductase (GSR), and the two subunits constituting the glutamate–cysteine ligase complex: the catalytic subunit (*GCLC*) and the modifier subunit (*GCLM*) [108]. NRF2 also participates in GSH maintenance by regulating the transcription of various ROS-detoxifying enzymes such as *CAT*, glutathione peroxidase 2 (*GPX2*), glutathione S-transferase (*GST*), N-acetyltransferase (*NAT*) and SOD [107,109,110]. NRF2 also regulates the TXN-based antioxidant system by regulating the expression of TXN, thioredoxin reductase 1 (*TXNRD1*), and sulfiredoxin (*SRXN1*) [111]. NRF2 further supports NADPH production by positively regulating principal NADPH-generating enzymes, like glucose-6-phosphate dehydrogenase (*G6PD*), 6-phosphogluconate dehydrogenase (*PGD*) and isocitrate dehydrogenase 1 (*IDH1*), in primary cortical astrocytes, lung cancer cells, and mouse small intestine and liver [107,112,113,114]. Another critical cytoprotective enzyme regulated by NRF2 is heme oxygenase (*HO1*), responsible for heme molecule breakdown [115]. NRF2 upregulates the expression of *HO1*, and ferritin light and heavy chains (*FTL* and *FTH*) to prevent hydroxyl radical formation by sequestering iron ions, thus inhibiting the Fenton reaction [116,117].

### 3.2. Mitochondrial Function and Biogenesis

Beyond its crucial role in ATP production, mitochondria are susceptible to damage and ROS generation. NRF2 emerges as a key factor in maintaining mitochondrial function. NRF2 and phosphoglycerate mutase 5 (PGAM5) are required for mitochondrial retrograde trafficking. At the basal level, NRF2 is associated with KEAP1 and PGAM5 to form PGAM5-KEAP1-NRF2 complex, and either of them will be dissociated from KEAP1 in response to oxidative stress [118]. NRF2 influences mitochondrial function directly by regulating the expression of critical enzymes for electron transport chain, and contributes to mitochondrial biogenesis by regulating sirtulin 1 (*SIRT1*), peroxisome proliferator-activated receptor-γ (*PPARG*), mitochondrial transcription factor A (*TFAM*), nuclear respiratory factor 1 (*NRF1*), and peroxisome proliferator-activated receptor γ coactivator 1α (*PPARGC1A*) [119,120,121,122,123]. NRF2-deficient neural cells are susceptible to chemically induced mitochondrial damage, while NRF2 overexpression protects cells against these injuries [124,125].

### 3.3. Inflammation

Microglia and astrocytes activate ARE genes to reduce oxidative stress and inflammation [126,127]. NRF2 activation by oxidative stress can block the NFκB pathway through the induction of antioxidant genes, subsequently reducing proinflammatory cytokine production [123,128]. However, the anti-inflammatory effect of NRF2 goes beyond simply reducing oxidative stress. NRF2 can directly regulate the expression of anti-inflammatory mediators like interleukin 17D (*IL17D*) and cluster of differentiation 36 (*CD36*), further solidifying its role in this process [107,129,130]. Moreover, NRF2 has been shown to suppress proinflammatory cytokines like tumor necrosis factor α (TNF-α), interleukin-6 (IL-6), and interleukin-1β (IL-1β) [131,132]. 

One important mechanism underlying this interplay is the counterbalance between the NRF2 and NFκB pathways. P65, a protein regulating both pathways, plays a key role. Upon inflammatory pathway activation, GSK-3 phosphorylates P65, leading to the degradation of the inhibitory protein inhibitor of NFκB α (IkBα) [133]. This enables the P65 to translocate to the nucleus and initiate NFκB transcription [133]. Notably, P65 also binds to KEAP1 in the nucleus to facilitate NRF2 dissociation from the ARE region, promoting its export and degradation [134]. Moreover, P65 inhibits NRF2 activity by competitive association with cAMP response element-binding protein (CREB)-binding protein (CBP) [135], which is required for the transactivation of NRF2 [79]. On the other hand, HO1, the downstream protein of NRF2, inhibits the translocation of P65 [123]. Therefore, this regulatory loop is crucial for controlling the inflammatory response.

### 3.4. Autophagy and Mitophagy

Macroautophagy, a conserved process essential for cellular survival, degrades long-lived proteins, removes damaged organelles, and clears protein aggregates [136]. Excessive ROS stimulate autophagy and increase autophagosome formation [137,138,139]. A fascinating link between autophagy and NRF2 is P62, a protein that serves both as an autophagy substrate and a cargo receptor [140,141,142]. P62 interacts with KEAP1 at NRF2-binding site, leading to subsequent NRF2 release and its nuclear translocation (Figure 1) [142]. Furthermore, oxidative stress also upregulates P62 expression through NRF2 and ARE, creating a positive feedback loop [62]. 

### 3.5. Endoplasmic Reticulum Stress and Unfolded Protein Response

The accumulation of misfolded proteins, such as polyglutamine aggregation, within the endoplasmic reticulum (ER) triggers ER stress and activates the unfolded protein response (UPR) [143]. The UPR employs conserved signaling pathways to restore cellular homeostasis. In cases of persistent ER stress, UPR triggers apoptosis to prevent further damage. Protein kinase RNA-like endoplasmic reticulum kinase (PERK), a key UPR signaling molecule, phosphorylates eukaryotic initiation factor 2α (EIF2α), leading to reduced protein synthesis and promoting cell survival during ER stress [143]. ROS within the ER tightly regulate the UPR response. Notably, ER-resident peroxidases 4, GSH peroxidase 7 (GPX7) and 8 (GPx8) facilitate H_2_O_2_ detoxification by catalyzing electron transfer from protein disulfide isomerase-like oxidoreductases [144,145,146]. PERK-mediated phosphorylation of NRF2 and promote the expression of ARE genes that increase GSH levels, reduce ER-associated ROS, activate transcriptional networks for mitochondrial biogenesis, and enhance cell survival [147,148,149]. Tumor-associated myeloid-derived suppressor cells lacking PERK exhibited impaired NRF2-driven antioxidant capacity and disrupted mitochondrial respiratory homeostasis [150]. Furthermore, NRF2 upregulates the expression of ER-resident antioxidant enzyme *GPX8* [151], further supporting its role in mediating ER stress.

## 4. Implication of NRF2 in Trinucleotide Repeat Disorders

In trinucleotide repeat expansion disorders, the cellular pathways protecting against oxidative stress often become dysregulated, leading to an inadequate response to ROS overload [131,152,153]. The NRF2 pathway appears defective in trinucleotide repeat expansion disorders [131,153], while the modulation of NRF2 signaling has shown beneficial effects in haltering these degenerations (Table 2).

### 4.1. NRF2 and Huntington’s Disease

Huntington’s disease (HD), a progressive, inherited neurodegenerative disease affecting the striatum, cerebral cortex, and thalamus [182,183], arises from an abnormal expansion of the CAG trinucleotide repeat in the huntingtin (*HTT*) gene [17]. This expansion, present in healthy individuals in less than 34 repeats, grows to 35–140 repeats in HD patients [184]. This mutant huntingtin (mHTT) triggers a detrimental cascade involving protein aggregation, altered gene expression, mitochondrial deficits, chronic inflammation, and oxidative stress, ultimately leading to progressive motor dysfunction, psychiatric disturbance, cognitive decline, and dementia within 15–20 years of symptom onset [185,186,187,188,189].

Among the various pathogenic mechanisms implicated in HD, oxidative stress and inflammation are particularly noteworthy and serve as potential therapeutic targets. Brain tissue from HD patients shows mitochondrial DNA damage, diminished levels of oxidative phosphorylation enzymes, and iron-mediated mitochondrial impairment [152,190]. Moreover, HD patients exhibit elevated levels of peroxidative molecules, like malondialdehyde (MDA) and 8-hydroxy-deoxyguanosine (8-OHdG), coupled with reduced levels of protective antioxidant proteins, such as GSH, GPX, and SOD1, in peripheral blood [191,192]. Studies on STHdh^Q111/Q111^ HD transgenic mouse models demonstrate reduced NRF2 activity and altered expression of KEAP1 and P62 in striatal cells [193]. Overexpression of mutant HTT in PC12 cells affects the expression of NRF2 and its responsive proteins such as NQO1, GCLC, TXNRD1, GSTA4, and GSTA6 [194]. Neural stem cells derived from HD patients display heightened susceptibility to oxidative stress, with correction of the disease-causing mutation restoring cellular redox balance [154]. 

Numerous NRF2 inducers show potential in alleviating neurodegeneration in HD. The natural NRF2 inducer sulforaphane (SFN) exhibited potential by enhancing mHTT protein degradation and improving cell viability in HEK293 cells overexpressing *HTT* with 94 CAG repeats [154]. Moreover, SFN has demonstrated efficacy in ameliorating motor dysfunction and reducing striatal cell death in HD mice induced by 3-nitropropionic acid (3-NP) [155]. The triterpenoid derivative 2-cyano-3,12-dioxooleana-1,9-dien-28-oic acid-methyl amide (CDDO-MA) reduced neuronal loss, MDA and 8-OHdG in the striatum by activating NRF2 in 3-NP-induced rats [156]. Similar CDDO derivatives, CDDO-ethyl amide (CDDO-EA) and CDDO-trifluoroethyl amide (CDDO-TFEA), upregulate NRF2/ARE-regulated genes, alleviate oxidative stress, improve motor function, and enhance survival in N171-82Q HD transgenic mice [157]. Dimethyl fumarate (DMF), an orally bioavailable NRF2 inducer, showed beneficial effects on survival time, motor function, and preservation of neurons in the striatum and motor cortex in two transgenic HD mouse models, YAC128 and R6/2 [158]. Naringin, a flavanone found in grapefruit and citrus species, reduced 3-NP-induced neuronal loss, ROS, and inflammation through NRF2 activation in the striatum of rats [159]. Protopanaxtriol, a constituent of Panax ginseng Meyer, decreased ROS production, enhanced nuclear translocation, and expression of HO1 and NQO1 in the striatum, while also improving motor function in 3-NP-induced HD rats [162]. Gintonin, a ginseng-derived lysophosphatidic acid receptor ligand, mitigated neurological impairment severity and lethality in 3-NP-induced mice by promoting nuclear translocation of NRF2 [163]. Harmine, a plant-derived β-carboline alkaloid, increased the expression of NRF2, HO1, NQO1 and P62, and enhanced motor and cognitive functions in 3-NP-induced HD rats [165]. Diapocynin upregulated the expression of NRF2, GST, GSH, NFκB, and improved motor function in 3-NP-induced HD rats [164]. Luteolin and its synthetic derivatives, Lut-C4 and Lut-C6, upregulated the expression of NRF2, SOD1 and GCLC, and improved cell viability in striatal cells from STHdh^Q111/Q111^ HD transgenic mice [160]. Resveratrol also activated the NRF2 pathway [195] and alleviated motor dysfunction in YAC128 mice [161]. The triazole-containing NRF2 inducer 5-nitro-2-{[5-(phenoxymethyl)-4-phenyl-4H-1,2,4-triazol-3-yl]thio}pyridine (MIND4-17) increased NQO1 and GCLM expression in neural stem cells differentiated from HD patient-derived induced pluripotent stem cells [131]. In STHdh^Q111/Q111^ mice, miR-196a facilitated NRF2 nuclear translocation by downregulating ubiquitin-specific peptidase 15 (USP15), a deubiquitin enzyme involved in the KEAP1–CUL3–RBX1 complex [196].

### 4.2. NRF2 and Spinocerebellar Ataxia

Spinocerebellar ataxias (SCAs) caused by CAG repeat expansions (SCA1, 2, 3, 6, 7, 17 and DRPLA) are characterized by progressive degeneration of the cerebellum, leading to impaired coordination and gait [197]. This degeneration primarily affects Purkinje and granule cell layers, as well as neurons in the deep cerebellar nuclei [198,199]. The neurodegeneration extends beyond the cerebellum to affect brainstem, basal ganglia, spinal cord, and even peripheral nerves in various SCA forms [198,200,201]. A common pathological hallmark of SCAs is the formation of toxic protein aggregates by proteins carrying expanded polyglutamine tracts [200]. Initially, these aggregates may manifest as small oligomers, thought to be even more toxic to cells than larger inclusions [198]. These aggregates disrupt critical cellular processes and lead to an imbalance between the production of ROS and the cellular antioxidant defense system, particularly in Purkinje neurons, which are known to have high energy demands [166].

#### 4.2.1. SCA1

SCA1 is caused by abnormal polyglutamine expansions in the ataxin-1 (ATXN1), which interacts with proteins that control gene transcription and splicing [202]. In *ATXN1-154Q* knock-in mice for SCA1, the mutant ATXN1 disrupts the function of the high mobility group box 1 complex (*HMGB1*), leading to increased mitochondrial DNA damage [203,204]. These mice exhibit morphological alterations in mitochondria, dysfunctional enzymes for electron transport chain, and elevated oxidative stress [166]. Interestingly, treating pre- or early symptomatic *ATXN1*-*154Q* knock-in mice with MitoQ, a compound that promotes NRF2 activation and translocation to the nucleus, reduced the levels of 8-OHdG and Purkinje cell loss and delayed the onset of motor coordination impairments [166]. 

#### 4.2.2. SCA2

Studies using fibroblasts from SCA2 patients demonstrated impaired mitochondrial network structure, altered expression and activity of antioxidant genes, increased production of ROS, and decreased activity of complexes I, II, and III of the electron transport chain [167]. Coenzyme Q10, a lipid-soluble vitamin-like benzoquinone compound that plays an important role in the mitochondrial respiratory chain, exerted antioxidant and anti-apoptotic functions by upregulating the expression of NRF2, NQO1, SOD and GSH [205]. Although treatment with coenzyme Q10 partially ameliorates these defects in the fibroblasts of SCA2 patients [167], its clinical efficacy in human trials remains uncertain. A study investigating SCA1, SCA2, SCA3, and SCA6 found beneficial effects of coenzyme Q10 on clinical progression only in patients with SCA1 and SCA3 over a two-year follow-up period [206]. The treatment did not appear to significantly impact disease deterioration in patients with SCA2 or SCA6 [206].

#### 4.2.3. SCA3

SCA3, the most common and well-studied SCA, highlights the connection between oxidative stress and neurodegeneration. SH-N-SH cells expressing ataxin-3 (*ATXN3*) with 78 CAG repeats showed reduced cell viability, decreased levels of GSH, and reduced expression of GSR, SOD, and CAT [207]. Reduced activity of complex II in the electron transport chain has also been reported in PC6-3 cells expressing *ATXN3* with 108 CAG repeats. Cerebellar granular cells from *ATXN3-71Q* transgenic mouse models, as well as in lymphoblastic cell lines from SCA3 patients, showed increased ROS generation [208]. Clinical studies consistently reveal reduced levels of GSH and TXN, as well as increased mitochondrial DNA damage and reduced mitochondrial copy numbers in the blood of SCA3 patients [207,209]. A large study consistently revealed increased ROS levels and reduced SOD and GPX activities in the blood of SCA3 patients, with these changes also correlating with disease severity [210].

Studies indicate that ATXN3 aggregation reduces NRF2 levels and activity [169,170]. NRF2 activator ASC-JM17 and DMF upregulated CAT, GSH, NQO1, HO1, SOD1, SOD2, and nuclear levels of NRF2, while reducing intracellular aggregates, enhancing cell viability and improving mitochondrial function in SK-N-SH cells expressing *ATXN3* with 78 CAG repeats [168]. Interestingly, treatment with aqueous extract of *Gardenia jasminoides* or *Glycyrrhiza inflata* reduced ROS levels and improved cell viability by upregulating NRF2, NQO1, GCLC, GST1, and SOD2 in HEK293 and SH-SY5Y cells expressing *ATXN3* with 75 CAG repeats [169,170]. Resveratrol enhanced the expression of NRF2, HO1, SOD, GPX, and P62 with reduced ROS levels, increased autophagy activity, and improved mitochondrial function and cell viability in SK-N-SH cells and *Drosophila* expressing *ATXN3* with 78 CAG repeats [117].

#### 4.2.4. SCA7

In PC12 cells expressing ataxin-7 (*ATXN7*) with 65 CAG repeats, the mutant ATXN7 increased ROS levels and disrupted the normal function of NADPH oxidase complexes [171]. Treatment with two well-known NRF2 inducers, N-acetylcysteine and vitamin E, reduced ROS levels and ATXN7 aggregation in an inducible SCA7 cell model [171]. These findings suggest that targeting the NRF2 pathway may be a therapeutic strategy for SCA7.

#### 4.2.5. SCA17

In *TBP-71Q* and *TBP-105Q* transgenic mice, an expanded polyglutamine tract in the TATA-binding protein (TBP) led to decreased expression levels of heat-shock protein β1 (HSBP1), a molecular chaperone known to protect cells from oxidative stress [211]. Furthermore, lymphoblastoid cells from SCA17 patients displayed increased susceptibility to oxidative stress [212] and downregulated expression of NQO1 and HO1 [172]. Treatment with the NRF2 inducers resveratrol or genipin improved cell viability, reduced ROS levels, and restored the expression of NQO1 and HO1 [172]. Treatment with 3-Benzoyl-5-Hydroxy-2H-Chromen-2-One (LM-031) promoted neurite outgrowth and reduced aggregation, partially by enhancing NRF2 expression in SH-SY5Y cells expressing *TBP* with 79 CAG repeats [173]. Shaoyao Gancao Tang (SG-Tang), a formulated Chinese herbal medicine made of *P. lactiflora* and *G. uralensis*, inhibited aggregation and rescued motor deficits in a *TBP-109Q* transgenic mouse model via increasing NRF2 expression [174].

### 4.3. NRF2 and Spinobulbar Muscular Atrophy (SBMA)

Spinobulbar muscular atrophy (SBMA), also known as Kennedy’s disease, is an X-linked neuromuscular disorder characterized by the progressive loss of motor neurons in the spinal cord and brainstem [213,214]. While primarily affecting motor function, SBMA patients may also experience gynecomastia (enlarged breast tissue in males) and sensory loss [215,216]. SBMA is caused by an expansion of CAG repeats in the androgen receptor (*AR*) gene [16,217]. These expanded polyglutamine tracts result in an increase in the α-helix structure, retain some function in AR, and may alter specific protein–protein interactions [218]. The pathogenic mechanism underlying SBMA involves oxidative stress [153] and mitochondrial dysfunction [219]. The normal AR protein regulates the expression of mitochondrial proteins coded by both the nucleus and mitochondria [220]. However, motor neuron-derived (MN-1) expressing *AR* with 113 CAG repeats exhibited elevated levels of ROS, decreased mitochondrial mass and number, downregulated expression of PPARGC1A, TFAM, SOD, and CAT, and activated the apoptosis pathway [153]. Aggregates formed by AR with 48 CAG repeats sequestered mitochondria [221] and impaired mitochondrial transport along neurites in HeLa and NSC34 cells [222].

Reduced NRF2 expression is consistently observed in motor neurons of AR100Q transgenic mice for SBMA, resulting in the downregulation of SOD, NQO1, and GPX [223]. Curcumin, a natural antioxidant found in turmeric, has been shown to decrease the formation of misfolded aggregates and upregulate NRF2 [224,225,226,227,228]. ASC-JM17, a curcumin analog, upregulated NRF2, NQO1, HO1 and GCLC, as well as improved motor function and muscle wasting in AR97Q mice for SBMA [175]. A current first-in-patient randomized, double-blind, placebo-controlled Phase 1/2a study in SBMA patients is under progress to assess the safety, pharmacokinetic and pharmacodynamic effects of ASC-JM17 [229]. Another analog, ASC-J9, ameliorated AR aggregates, motor function, and muscle wasting in AR97Q mice [176]. Taken together, these findings suggest that curcumin analogs have potential as therapeutic agents in SBMA by activating the NRF2 signaling pathway.

### 4.4. NRF2 and Friedreich Ataxia

Friedreich’s ataxia (FRDA) is an autosomal recessive neurodegenerative disease resulting from a homozygous GAA trinucleotide repeat expansion within the first intron of the frataxin (*FXN*) gene [20,230]. These expanded GAA repeats induce histone deacetylation and aberrant DNA conformation, consequently leading to diminished mRNA levels and protein expression of FXN [231]. Clinically, FRDA manifests as progressive ataxia, diabetes, cardiomyopathy, skeletal abnormalities, and disruptions in both the central and peripheral nervous systems, with characteristic lesions observed in dorsal root ganglia, the dentate nuclei of the cerebellum and corticospinal tracts, along with the sensory peripheral nerves [232,233,234]. Although the precise function of FXN remains unclear, it is known to play roles in iron–sulfur cluster biogenesis and heme biosynthesis. FXN deficiency results in mitochondrial iron accumulation, triggering oxidative stress in the affected tissues [235,236,237,238,239,240,241].

Lines of evidence have elucidated an impairment of the NRF2 pathway in FRDA [242,243,244,245]. Elevated levels of oxidative damage to both nuclear and mitochondrial DNA have been identified in the peripheral blood cells of FRDA patients, coupled with increased plasma MDA and urine 8-OHdG levels [246,247]. Erythrocytes from FRDA patients demonstrated decreased levels of reduced GSH [248,249]. Omaveloxolone, an NRF2 activator, works by preventing the ubiquitination-mediated degradation of NRF2 [250,251]. A large, randomized placebo-controlled clinical trial demonstrated that omaveloxolone treatment significantly improved neurological deficits in FRDA patients [178], leading to its approval as the first treatment for FRDA by the US Food and Drug administration in 2023. Other NRF2 activators, such as SFN, DMF, N-acetylcysteine and vatiquinone (EPI-743), increased NRF2 expression, rebalanced the GSH/GSSG ratio, and upregulated FXN expression in fibroblasts and lymphoblastoid cells from FRDA patients [179,180]. Vatiquinone is an orally bioavailable compound that readily crosses the blood–brain barrier, with a no-observable-adverse-effect level of 100 mg/kg [252]. A phase 2 randomized, placebo-controlled trial demonstrated that vatiquinone significantly improved the neurological function in FRDA patients [181]. Treatment with vatiquinone also increased GSH levels in leukocytes and erythrocytes, and also enhanced the activity of GPX in erythrocytes [253].

### 4.5. NRF2 and Fragile X-Associated Tremor/Ataxia Syndrome

Fragile X syndrome is caused by a deficiency in the fragile X mental retardation 1 protein (FMR1) [254]. In most cases, this deficiency is caused by an expansion of CGG trinucleotide repeats in the FMR1 gene promoter, leading to transcriptional silencing [255]. More than 200 CGG repeats are associated with the classic fragile X syndrome, often characterized by features of intellectual disability and autism spectrum disorder [256]. CGG repeat expansions in the range of 55–200 within the *FMR1* gene cause fragile X-associated tremor/ataxia syndrome (FXTAS) [257,258]. FXTAS primarily affects adult males over 50 years old, with increasing penetrance with age [257,259]. Clinical manifestations of FXTAS include late-onset and progressive cerebellar ataxia, intention tremor, parkinsonism, cognitive decline, and peripheral neuropathy [257,259,260,261]. Interestingly, FXTAS patients typically show normal or low FMR1 protein levels, but elevated *FMR1* mRNA transcripts [262]. These transcripts accumulate in the nucleus of neurons and astrocytes, forming inclusions containing ubiquitin, a protein tag for degradation [262,263,264]. The long CGG expansions in *FMR1* mRNA may sequester other proteins, leading to the formation of dynamic intranuclear inclusions over time [42,265,266,267,268]. 

FMR1, a crucial RNA-binding protein that regulates mRNA metabolism, forms complexes with RNA and ribosomes, acting as a translation suppressor [269]. FMR1 is highly expressed in neurons, where it shuttles between the nucleus and neuronal processes [270,271]. Specifically at postsynaptic sites (where neurons receive signals), FMR1 transports mRNA cargo and its activity is tightly controlled by synaptic activity [272,273,274,275,276,277,278]. The absence of FMRP generally disrupts synaptic development and plasticity in specific brain regions [37,277,279]. Oxidative stress is a well-established feature of FXTAS, evidenced by increased lipid peroxidation, elevated levels of oxidative metabolites and ROS in both the fibroblasts and blood samples of FXTAS patients [280]. Dysregulated expression of mitochondrial proteins, including aconitase and ATPase β-subunit (ATPB), along with elevated ROS levels and diminished activities of complex I and IV in the electron transport chain, have been observed in the brain tissues of patients with FXTAS [281,282]. Moreover, reduced expression of manganese SOD in the frontal cortex of FXTAS patients has been shown [282]. Alterations in the mitochondrial network, along with impaired mitochondrial density and transport dynamics, have been reported in hippocampal neurons of preCGG KI mice for FXTAS and fibroblasts of FXTAS patients [283,284]. In fibroblasts from FXTAS patients, SFN has the potential to enhance pathways associated with brain function, bioenergetics, UPR, proteasome activity, antioxidant defenses, and iron metabolism, through NRF2-dependent and independent mechanisms [285]. 

## 5. NRF2 Activating Compounds

NRF2 activation, which can be induced by natural and synthetic compounds, has emerged as a promising therapeutic strategy for numerous diseases. Although initial research predominantly investigated NRF2 activators for cancer and inflammatory diseases, recent progress has yielded novel NRF2 activators tailored for the central nervous system. These compounds function by modulating the activity of KEAP1 protein, interfering with its interaction with NRF2, promote NRF2 nuclear accumulation, enhance PI3K/AKT, or elevating NRF2 expression-mediated by an unclear mechanism (Figure 2).

### 5.1. Curcumin and Its Derivatives

Curcumin, a natural compound found in turmeric, has been extensively studied for its diverse health benefits, including anti-inflammatory, anti-cancer, and antioxidant properties [286,287]. Its potential therapeutic applications extend to neurodegenerative diseases, with research indicating its ability to cross the blood–brain barrier and exert neuroprotective effects such as reducing oxidative stress and inflammation, enhancing energy metabolism, and modulating synaptic plasticity [288,289,290,291,292]. Studies have demonstrated that curcumin acts as a potent NRF2 inducer, thereby playing a neuroprotective role through the NRF2-ARE pathway [293,294,295]. The structural features of curcumin, including its two phenolic groups and β-diketone moiety, enable it to interact with KEAP1, which causes dissociation of NRF2 from KEAP1 and subsequent upregulation of antioxidant proteins [296,297,298,299,300]. Curcumin treatment in SH-SY5Y cells lowered ROS levels, boosted NRF2 expression, and enhanced cell survival against amyloid β toxicity [301]. Furthermore, curcumin treatment significantly improved motor function and restored the activity of tyrosine hydroxylase in a rotenone-induced rat model of Parkinson’s disease. This neuroprotective effect was probably mediated by the activation of the NRF2 pathway to restore HO1 and NQO1 expression [302].

The poor bioavailability of curcumin limits its clinical applications. Therefore, curcumin analogues that aim to retain beneficial properties while improving BBB penetration were developed. One such example is compound 28, which displays significant cell-protective effects and efficiently enters the brain following oral administration [303]. Other analogues, like ASC-JM17 and ASC-J9, have shown potential in treating SBMA [175,176]. Oral administration of ASC-JM17 enhanced cell viability and mitochondrial function in an SCA3 cell model [168]. Furthermore, studies have identified ASC-JM17 as a potent activator of key pathways involved in UPR, protein degradation, and anti-oxidative stress in cell, fly, and mouse models of SBMA [175]. Intraperitoneal injection of ASC-J9 reduced nuclear aggregation and improved cell survival in PC12 cells [176]. The administration of ASC-J9 to SBMA mice significantly mitigated muscular atrophy without notable alterations in serum testosterone levels, thus preserving normal sexual function and fertility [176].

### 5.2. Flavonoids

Flavonoids, a diverse group of phenolic compounds commonly found in plant-based foods and beverages, offer anti-neuroinflammatory and neuroprotective properties [304,305]. Within this family, many compounds have been shown to enhance the expression of the ARE gene [306,307]. For instance, naringenin has demonstrated the ability to mitigate neurotoxicity induced by 6-hydroxydopamine (6-OHDA) by upregulating NRF2 and activating the ARE pathway in SH-SY5Y cells [308,309]. Similarly, quercetin has been implicated in protecting against manganese-induced neurotoxicity by upregulating NRF2 and HO-1 expression in SK-N-MC cells and rats [310]. Quercetin stimulates NRF2-mediated ARE activity by inhibiting the degradation of NRF2 and enhancing the turnover of KEAP1 [311]. Quercetin also elevates the expression of NRF2 and its downstream antioxidative genes by enhancing the PI3K/AKT pathway [312].

Open-chain flavonoid chalcones, characterized by a three-carbon α,β-unsaturated carbonyl system, also display diverse biological properties, including anti-inflammation and antioxidant effects [313]. The chalcone derivative cardamonin binds to the cysteine residues or the Kelch domain of the KEAP1, causing NRF2 to dissociate from KEAP1 and translocate to the nucleus [314,315]. In addition, cardamonin also provides cytoprotection via activating NRF2 through the P62-dependent degradation of KEAP1 [316]. Licochalcone E, another chalcone, attenuates inflammatory responses in BV2 cells and protects SH-SY5Y cells from 6-OHDA cytotoxicity [317]. Oral administration of licochalcone E promotes nuclear translocation of NRF2 and activates the NRF2-ARE pathway, leading to increased GSH levels in mice treated with 1-methyl-4-phenyl-1,2,3,6-tetrahydropyridine [317].

### 5.3. Resveratrol

Resveratrol, also known as 3,5,4′-trihydroxytrans-stilbene, is found in foods, including berries, grapes, red wine, and peanuts [318,319,320,321]. This natural polyphenol exhibits a wide range of pharmacological effects, such as hepatoprotective, anti-diabetic, anti-cancer, antioxidant, anti-inflammatory, cardioprotective properties, and the potential to improve dyslipidemia [322,323,324,325,326,327]. The possible key molecular mechanism underlying the beneficial effects of resveratrol is by modulating the NRF2 pathway through the disruption of NRF2-KEAP1 binding and stimulation of the PI3K/AKT pathways [195]. It improved motor function in an HD mouse model [164]. It enhanced the expression of NRF2, HO1, SOD, and GPX, reduced ROS, improved mitochondrial function, and reduced neuronal death in SH-SY5Y cells expressing *ATXN3* with 78 CAG repeats [117]. Furthermore, resveratrol administration improved motor function in a SCA3 fly model by inducing NRF2 activation [117]. In lymphoblastoid cells derived from patients with SCA17, resveratrol upregulated the expression of NRF2, NQO1, and HO1, while reducing ROS levels [172]. However, the oral bioavailability for resveratrol is probably low [328].

### 5.4. Herb Extracts and Constituents

Herb extracts have gained attention for their potential therapeutic effects in treating neurodegenerative diseases due to their diverse bioactive compounds, which exhibit neuroprotective properties. Ginseng constituents, such as protopanaxtriol and gintonin, have been shown to reduce oxidative stress, improve motor function, and protect brain cells in HD rats [162,163,174]. Harmine, a β-carboline alkaloid in *Peganum harmala*, also improved motor and cognitive function in HD rats [165]. The herb extract from *Gardenia jasminoides*, along with its primary constituents genipin, geniposide, and crocin, has exhibited potential in inhibiting aggregation and mitigating oxidative stress in an SCA3 cell model [169]. Interestingly, genipin also reduced ROS levels in lymphoblastoid cells derived from SCA17 patients [172]. The extract of *Glycyrrhiza inflata* extract, and its constituents, licochalcone A and ammonium glycyrrhizinate, increased the expression of NRF2/ARE-related antioxidant proteins and reduced aggregation in an SCA3 cell model [170]. SG-Tang, a formulated herbal medicine comprising *Paeonia lactiflora* and *Glycyrrhiza uralensis*, decreased motor-deficits in a SCA17 mouse model [174]. The mode of action of these compounds is to enhance the expression of NRF2 and its downstream antioxidants, but how NRF2 expression is increased remains to be explored.

### 5.5. Sulforaphane

Sulforaphane (SFN), also known as 1-isothiocyanato-4-(methylsulfinyl)butane, is an aliphatic isothiocyanate derived from glucoraphanin, a precursor primarily found in cruciferous vegetables like broccoli, cauliflower, cabbage, and Brussels sprouts [329]. SFN acts as a sulfur-rich compound that was shown to increase NRF2 DNA-binding activity and disrupt the interaction of NRF2 and KEAP1 to upregulate the expression NQO1, HO1, GST, and TXNRD, thereby counteracting oxidative stress [330]. In a 3-NP-induced mouse model of HD, SFN upregulated NRF2, NQO1, GCL, and HO1, inhibited NFκB, TNF-α, IL-1β and IL-6 in the striatum, and improved motor dysfunction and reduced striatal cell death [155]. SFN also upregulated NRF2 and NQO1, promoting neurite growth in FXN-deficient motor neurons [179,180]. In fibroblasts from patients with FRDA, SFN normalized the expression of NRF2, NQO1, HO1, and GCL [179,180]. A proteomics study in fibroblasts from FXTAS patients demonstrated the potential of SFN to regulate bioenergetics, UPR, proteasome, antioxidant, and iron metabolism pathways [285]. However, SFN faces several biopharmaceutical challenges, such as poor aqueous solubility and low bioavailability [331]. Developing an appropriate drug delivery system for SFN could enhance its solubility and bioavailability.

### 5.6. Dimethyl Fumarate

Dimethyl fumarate (DMF) is an orally bioavailable fumaric acid ester that metabolizes into methyl hydrogen fumarate [332]. Originally used to treat psoriasis for decades, DMF has recently emerged as a therapy for multiple sclerosis [333]. DMF undergoes hydrolysis to its active metabolites, monomethyl fumarate (MMF) [334]. In addition to modifying the reactive cysteines in BTB domain, MMF binds to the β-propeller domain as well as the BTB domain of KEAP1 to free NRF2 from KEAP1, and thus activates the NRF2-ARE pathway [335]. DMF treatment in an HD mouse model upregulated NRF2 in the striatum, preventing weight loss, improving motor function, and extending survival [158]. Furthermore, DMF upregulated NRF2 and NQO1, while also increasing FXN levels in FRDA patient-derived fibroblasts and in FRDA mouse models [179,180].

### 5.7. Triterpenoid Derivatives

Triterpenoid, mainly derived from *Kadsura* genus, exerts anti-inflammatory, anticancer and anti-viral activities [336]. Their derivatives demonstrated the potential to inhibit the interaction between KEAP1 and NRF2 [337]. Synthetic triterpenoids, such as CDDO-MA, CDDO-EA, and CDDO-TFEA, activated NRF2/ARE-regulated genes, reduced oxidative stress in the stratum, and enhanced motor function and survival in rodent models of HD [156,157]. Omaveloxone, a synthetic triterpenoid, showed promising results in improving neurological deficits among FRDA patients in a large randomized–controlled clinical trial [178], and became the first FDA-approved treatment for FRDA. Oral administration of omaveloxolone at doses higher than 80 mg generated plasma concentrations consistent with those that significantly induced NRF2 target genes in non-human primates, as demonstrated by the pharmacokinetic/pharmacodynamic model developed in monkeys [338].

### 5.8. Fatty Acid Esters of Hydroxy Fatty Acids

Fatty acid esters of hydroxy fatty acids (FAHFAs) are a family of endogenous lipids consisting of a fatty acid esterified with a hydroxy fatty acid at various positions. The biosynthesis of FAHFAs, such as eicosapentaenoic acid esterified with 12-hydroxy stearic acid (12-EPAHSA) and docosahexaenoic acid esters of 12-hydroxy oleic acid (12-DHAHOA), upregulated NRF2 expression, thereby suppressing the oxidation of small lipid droplets and mitigating oxidative stress induced by H_2_O_2_ in C2A human hepatoma-derived cells [339,340]. The detailed mechanisms underlying FAHFA biosynthesis, bioavailability, pharmacokinetics, pharmacodynamics, and especially their role in NRF2 activation, remain to be elucidated.

## 6. Conclusions

Trinucleotide repeat expansion disorders exhibit impaired NRF2 signaling, resulting in insufficient responses to excessive ROS production. Studies have shown that NRF2 activators, such as curcumin, resveratrol, SFN, and DMF, as well as extracts from *Gardenia jasminoides* and *Glycyrrhiza inflata*, can effectively alleviate oxidative stress damage in cell and animal models of these disorders. However, translating these findings into successful clinical trials remains a challenge. This discrepancy might be attributed to the complexity of neurodegenerative mechanisms and the limitations in recruiting participants for studies on rare diseases. Given the multifaceted pathogenesis of these diseases, targeting multiple pathways simultaneously could offer a more effective approach. A deeper understanding of the specific disease mechanisms and the development of better biomarkers are also crucial. Bridging this gap between preclinical findings and effective clinical outcomes requires further research into the mechanisms of action of these NRF2 activators and their clinical efficacy.

## Figures and Tables

**Table 1:** Genetics and main clinical features of trinucleotide repeat expansion disorders.

Disease	Inheritance	Gene	Repeat Motif	Expanded Repeat Length	Location	Main Clinical Features
HD	Autosomal dominant	*HTT*	CAG	>35	CDS	Chorea, dystonia, dementia, psychosis
SCA1	Autosomal dominant	*ATXN1*	CAG	>38	CDS	Ataxia, spasticity, dementia
SCA2	Autosomal dominant	*ATXN2*	CAG	>31	CDS	Ataxia, polyneuropathy
SCA3	Autosomal dominant	*ATXN3*	CAG	>60	CDS	Ataxia, parkinsonism, spasticity
SCA6	Autosomal dominant	*CACNA1A*	CAG	>19	CDS	Ataxia, nystagmus
SCA7	Autosomal dominant	*ATXN7*	CAG	>36	CDS	Ataxia, retinitis pigmentosa
SCA17	Autosomal dominant	*TBP*	CAG	>46	CDS	Ataxia, seizures, dementia, psychosis
DRPLA	Autosomal dominant	*ATN1*	CAG	>48	CDS	Ataxia, chorea, seizure, dementia
SBMA	X-linked recessive	*AR*	CAG	>37	CDS	Muscle atrophy, dysphagia, gynecomastia, infertility
FRDA	Autosomal recessive	*FXN*	GAA	>200	Intron	Sensory ataxia, cardiomyopathy, diabetes
FXTAS	X-linked recessive	*FMR1*	CGG	60–200	5′UTR	Ataxia, tremor, parkinsonism, dementia
SCA8	Autosomal dominant	*ATXN8OS*	CTG	>74	3′UTR	Ataxia, dysarthria, nystagmus
SCA12	Autosomal dominant	*PPP2R2B*	CAG	>54	5′UTR	Ataxia, seizure

*AR*: androgen receptor; *ATN1*: atrophin-1; *ATXN1*: ataxin-1; *ATXN2*: ataxin-2; *ATXN3*: ataxin-3; *ATXN7*: ataxin-7; *ATXN8OS*: ataxin-8 opposite strand; *CACNA1A*: calcium voltage-gated channel subunit α 1A; CDS: coding sequence; DRPLA: dentatorubral-pallidoluysian atrophy; *FMR1*: fragile X mental retardation 1; FRDA: Friedreich ataxia; *FXN*: frataxin; FXTAS: fragile x-associated tremor/ataxia syndrome; HD: Huntington’s disease; *HTT*: huntingtin; *PPP2R2B*: protein phosphatase 2 regulatory subunit B β; SBMA: spinobulbar muscular atrophy; SCA: spinocerebellar ataxia; *TBP*: TATA-box binding protein; UTR: untranslated region.

**Table 2:** The efficacy of NRF2-activating compounds in treating trinucleotide repeat expansion disorders.

Disease	Compound	Cell Model	Animal Model	Clinical Study	Benefit	Reference
HD	Sulforaphane	HEK293 cells overexpressing *HTT* with 94 CAG repeats			Increase of HTT degradation and cell viability	[154]
			3-NP-treated mice		Decreased neurological impairment and lethality	[155]
	CDDO-MA		3-NP-treated rats		Reduction in neuronal loss in striatum	[156]
	CDDO-EA and CDDO-TFEA		N171-82Q mice		Improvement of motor function and survival	[157]
	DMF		YAC128 and R6/2 mice		Improvement of motor function and survival; preservation of neurons in the striatum and motor cortex	[158]
	Naringin		3-NP-treated rats		Reduction in neuronal loss, ROS and inflammation in striatum	[159]
	Luteolin, Lut-C4, Lut-C6	Striatal cells from STHdh^Q111/Q111^ HD transgenic mice			Improvement of cell viability	[160]
	Resveratrol		YAC128 mice		Improvement of motor function	[161]
	MIND4-17	Neural stem cells from HD-iPSCs			Increases the expression of NQO1 and GCLM	[131]
	Protopanaxtriol		3-NP-treated rats		Reduction in ROS in the striatum, improvement of motor function	[162]
	Gintonin		3-NP-treated mice		Improvement of motor function and survival	[163]
	Diapocynin		3-NP-treated rats		Improvement of motor function	[164]
	Harmine		3-NP-treated rats		Improvement of motor and cognitive functions	[165]
SCA1	MitoQ		ATXN1-154Q mice		Reduction in Purkinje cell loss; delay of the onset of motor impairment	[166]
SCA2	Coenzyme Q10	Fibroblasts of SCA2 patients			Reduction in ROS	[167]
SCA3	ASC-JM17	SK-N-SH cells expressing *ATXN3* with 78 CAG repeats			Improvement of cell viability; reduction in aggregation	[168]
	DMF	SK-N-SH cells expressing *ATXN3* with 78 CAG repeats			Improvement of cell viability; reduction in aggregation	[168]
	*Gardenia jasminoides*	HEK293 and SH-SY5Y cells expressing *ATXN3* with 75 CAG repeats			Reduction in ROS; improvement of cell viability	[169]
	*Glycyrrhiza inflata*	HEK293 and SH-SY5Y cells expressing *ATXN3* with 75 CAG repeats			Reduction in ROS; improvement of cell viability	[170]
	Resveratrol	SK-N-SH cells expressing *ATXN3* with 78 CAG repeats			Reduction in ROS; improvement of cell viability	[117]
SCA7	N-acetylcysteine	PC12 cells expressing *ATXN7* with 65 CAG repeats			Reduction in ROS and aggregation	[171]
	Vitamin E	PC12 cells expressing *ATXN7* with 65 CAG repeats			Reduction in ROS and aggregation	[171]
SCA17	Resveratrol	lymphoblastoid cells from SCA17 patients			Improvement of cell viability; reduction in ROS	[172]
	Genipin	lymphoblastoid cells from SCA17 patients			Improvement of cell viability; reduction in ROS	[172]
	LM-031	SH-SY5Y cells expressing *TBP* with 79 CAG repeats			Reduction in aggregation	[173]
	SG-Tang	SH-SY5Y cells expressing *TBP* with 79 CAG repeats			Reduction in aggregation; increased neurite outgrowth	[174]
			TBP-109Q mice		Reduction in aggregation and improvement of motor function	[174]
SBMA	ASC-JM17		AR97Q mice		Improvement of motor function and muscle wasting	[175]
	ASC-J9		AR97Q mice		Improvement of motor function and muscle wasting	[176]
FRDA	Omaveloxolone	Cerebellar granular neuronss from KIKO and YG8R mice			Restoration of complex I activity.	[177]
		Skin ffibroblasts from FRDpatients			Reductopm of lipid peroxidation and mitochondrial ROS, and upregulation of GSH	[177]
				randomized placebo-controlled clinical trial	Improvement of neurological deficits	[178]
	Sulforaphane	Lymphoblastoid cells from FRDA patients			Upregulation of *FXN* expression	[179,180]
	DMF	Lymphoblastoid cells from FRDA patients			Upregulation of *FXN* expression	[180]
	N-acetylcysteine	Lymphoblastoid cells from FRDA patients			Upregulation of *FXN* expression	[180]
	Vatiquinone (EPI-743)	Lymphoblastoid cells from FRDA patients			Upregulation of *FXN* expression	[180]
				randomized placebo-controlled clinnical trial	Improvement of neurological deficits	[181]

CDDO-EA: 2-cyano-3,12-dioxooleana-1,9-dien-28-oic acid-ethyl amide; CDDO-MA: 2-cyano-3,12-dioxooleana-1,9-dien-28-oic acid-methyl amide; CDDP-TFMA: 2-cyano-3,12-dioxooleana-1,9-dien-28-oic acid-trifluoroethyl amide; DMF: dimethyl fumarate; FRDA: Friedreich’s ataxia; GCLM: glutamate-cysteine ligase modifier subunit; HD: Huntington’s disease; HD-iPSCs: induced pluripotent stem cells derived from Huntington’s disease patients; HTT: huntingtin; LM-031: 3-Benzoyl-5-Hydroxy-2H-Chromen-2-One; MIND4-17: 5-nitro-2-{[5-(phenoxymethyl)-4-phenyl-4H-1,2,4-triazol-3-yl]thio}pyridine; 3-NP: 3-nitropropionic acid; ROS: reactive oxygen species; NQO1: NAD(P)H quinone dehydrogenase 1; SBMA: spinobulbar muscular atrophy; SCA: spinocerebellar ataxia; SG-Tang: Shaoyao Gancao Tang.
